# Upregulation of YKL-40 Promotes Metastatic Phenotype and Correlates with Poor Prognosis and Therapy Response in Patients with Colorectal Cancer

**DOI:** 10.3390/cells11223568

**Published:** 2022-11-11

**Authors:** Mariangela De Robertis, Maria Raffaella Greco, Rosa Angela Cardone, Tommaso Mazza, Flaviana Marzano, Nikolay Mehterov, Maria Kazakova, Nikolay Belev, Apollonia Tullo, Graziano Pesole, Victoria Sarafian, Emanuela Signori

**Affiliations:** 1 Department of Biosciences, Biotechnology and Biopharmaceutics, University of Bari ‘A. Moro’, 70125 Bari, Italy; 2 Unit of Bioinformatics, Fondazione IRCCS Casa Sollievo della Sofferenza, 71013 San Giovanni Rotondo, Italy; 3 Institute of Biomembranes, Bioenergetics and Molecular Biotechnologies, Consiglio Nazionale delle Ricerche, 70126 Bari, Italy; 4 Department of Medical Biology, Medical University of Plovdiv, 4000 Plovdiv, Bulgaria; 5 Research Institute at Medical University of Plovdiv, 4000 Plovdiv, Bulgaria; 6 University Hospital Eurohospital, 4000 Plovdiv, Bulgaria; 7 Department of Propedeutics of Surgical Diseases, Medical University of Plovdiv, 4000 Plovdiv, Bulgaria; 8 Laboratory of Molecular Pathology and Experimental Oncology, Institute of Translational Pharmacology, Consiglio Nazionale delle Ricerche, 00133 Rome, Italy

**Keywords:** colorectal cancer, biomarkers, YKL-40, metastasis

## Abstract

YKL-40 is a heparin- and chitin-binding glycoprotein that belongs to the family of glycosyl hydrolases but lacks enzymatic properties. It affects different (patho)physiological processes, including cancer. In different tumors, YKL-40 gene overexpression has been linked to higher cell proliferation, angiogenesis, and vasculogenic mimicry, migration, and invasion. Because, in colorectal cancer (CRC), the serological YKL-40 level may serve as a risk predictor and prognostic biomarker, we investigated the underlying mechanisms by which it may contribute to tumor progression and the clinical significance of its tissue expression in metastatic CRC. We demonstrated that high-YKL-40-expressing HCT116 and Caco2 cells showed increased motility, invasion, and proliferation. YKL-40 upregulation was associated with EMT signaling activation. In the AOM/DSS mouse model, as well as in tumors and sera from CRC patients, elevated YKL-40 levels correlated with high-grade tumors. In retrospective analyses of six independent cohorts of CRC patients, elevated YKL-40 expression correlated with shorter survival in patients with advanced CRC. Strikingly, high YKL-40 tissue levels showed a predictive value for a better response to cetuximab, even in patients with stage IV CRC and mutant KRAS, and worse sensitivity to oxaliplatin. Taken together, our findings establish that tissue YKL-40 overexpression enhances CRC metastatic potential, highlighting this gene as a novel prognostic candidate, a predictive biomarker for therapy response, and an attractive target for future therapy in CRC.

## 1. Introduction

Colorectal cancer (CRC) is the third leading cause of cancer death and the fourth most commonly diagnosed cancer in the world [1]. Despite the increasing access to guideline-recommended screening for early detection and the continuous advancements in high-quality treatment options, approximately 25% of patients at initial diagnosis show metastases, and 20% of the remaining cases may develop metastatic CRC (mCRC), which is the leading cause of CRC-related mortality [2]. Current clinical-stage classification provides the main information for CRC patient risk stratification and the selection of the most appropriate therapy. Nevertheless, the heterogeneous nature of tumors and the varying responses to different treatments often cause diverse patient outcomes, even within the same prognostic group [3]. In addition, drug resistance remains a major barrier to effective cancer treatment, including in targeted therapy approaches [4]. Several molecular alterations, such as functional mutations of BRAF, PI3KCA, PTEN, PDGFRA, or MEP2K1 and amplifications of c-MET, ERBB2, and FGFR1, have been related to resistance to anti-EGFR target therapy [5,6,7,8]. For example, cetuximab and panitumumab, two anti-EGFR monoclonal antibodies, both FDA-approved for the first-line treatment of CRC, are more effective in wild-type (WT)-RAS patients, although 40–60% of them are still non-responders [9].

Furthermore, the “kinome reprogramming mechanism” can be involved in therapy resistance under pharmacologic pressure. Through the activation of compensatory kinases, tumor cells circumvent the action of kinase-targeted cancer therapies, facilitating therapeutic resistance [10,11,12].

Robust prognostic biomarkers to better stratify patients at high/low CRC risk and predictive biomarkers of the response to treatment are currently lacking. To bridge this gap, many efforts have been made to identify new accurate biomarkers that can also serve as potential drug targets. Numerous CRC candidate biomarkers have emerged in the last few decades, such as diagnostic, prognostic, and therapy-response predictive molecules, both tissue and blood-based. They include the detection of proteins [13], such as the c-MYC protein (a downstream effector of many oncogenic kinases involved in the “kinome reprogramming phenomena”) [14,15,16,17], epigenetically modified tumor DNA [18,19,20], and microRNAs (miRs) that are often tightly linked to proliferation, metastasis, and drug resistance [21,22], such as miR-31, miR-143, and miR-145, that could have a pivotal role in the resistance to anti-EGFR treatment in mCRC [23,24].

Recently, Chitinase-3-like protein 1 (CHI3L1), also known as YKL-40, has aroused much interest as a circulating candidate biomarker and therapeutic target in CRC [25,26]. YKL-40 is a 40 kDa heparin- and chitin-binding glycoprotein that belongs to the glycosyl hydrolase family but lacks enzymatic properties [27].

The glycoprotein is produced by a variety of cells, including macrophages, neutrophils, synoviocytes, chondrocytes, fibroblast-like cells, endothelial cells, vascular smooth muscle cells, hepatic stellate cells, and cancer cells [28]. Recently, a few receptors have been identified. YKL-40 participates in a multimeric complex with IL-13-IL-13Rα2 and synergistically interacts with TMEM219 and Gal-3 [29]. Furthermore, Geng et al. revealed that YKl-40 physically interacts with CD44 to activate the Erk and Akt pathways [30].

YKL-40 dysregulation has been related to chronic inflammatory diseases and cancer [31,32,33]. The molecular mechanisms of this phenomenon still need to be clarified. A number of studies have verified the association of increased YKL-40 expression with rheumatoid arthritis, osteoarthritis, asthma, inflamed tissues in ulcerative colitis, and Crohn’s disease [34]. It is reported that pro-inflammatory cytokines such as IL-1, TNF-α, IL-6, and IL-13 regulate YKL-40 expression in these conditions [35].

In different tumors, YKL-40 gene overexpression is linked to higher cell proliferation, angiogenesis, and vasculogenic mimicry, migration, and invasion. This supports its possible use as a novel therapeutic target, as recently suggested by preclinical studies using an anti-YKL-40 antibody in brain tumors [36].

The ability of YKL-40 to interact with fibronectin, collagens I, II, III, and IV, and hyaluronic acid is especially important for cancer cell migration and invasion [37,38,39].

Regarding CRC, an increasing number of studies are highlighting the property of the serological YKL-40 level to serve as a risk predictor and as an independent prognostic biomarker [40]. However, its pathogenic role and prognostic value in patients with mCRC have not yet been investigated. Intending to elucidate novel and still unexplored YKL-40 functions linked to CRC progression and drug resistance, we studied the relationship between YKL-40 and metastatic potential in CRC. The migration, invasion, and proliferation of two CRC lines subjected to forced YKL-40 down-/overexpression were determined and compared. The role of YKL-40 in regulating EMT-marker genes was assessed. Then, YKL-40 levels were measured in tumor tissues and serum samples of CRC patients, as well as in the early and late tumor phases of a CRC mouse model. Finally, the clinical relevance of its tissue expression level was assessed in patients with mCRC (GEO and TCGA databases) to define its prognostic role and predictive value for responsiveness to cetuximab and oxaliplatin.

## 2. Materials and Methods

### 2.1. Cell Culture

The human CRC cell lines HCT116 (KRASmut and p53-WT) and Caco2 (KRAS-WT and p53mut) were grown in DMEM supplemented with 10% FCS, 2 mM L-glutamine, and 100,000 U/L Penicillin/Streptomycin (Thermo Fisher Scientific, Waltham, MA, USA). All cell lines were cultured at 37 °C with 5% CO_2_.

### 2.2. Modulation of YKL-40 Gene Expression

The experiments were performed considering the following treatment groups: YKL-40 knockdown cells (YKL-40.KD), pCMV3-YKL-40 cells (YKL-40.OE), pCMV3-YKL-40_KD cells (YKL-40.KD rescued), and untreated control cells (YKL-40.WT). Cells treated only with transfection reagents were used as technical control groups. YKL-40 knockdown was generated using CRISPR/Cas9 technology. Cells were transfected with TrueGuide Synthetic crRNA CRISPR974196_CR annealed with tracrRNA using Lipofectamine Cas9 Plus Reagent and Lipofectamine CRISPRMAX Reagent (Thermo Fisher Scientific) according to the manufacturer’s protocol. TrueGuide crRNA Positive Control HPRT1 and TrueGuide crRNA Negative Control were used as positive and negative controls, respectively. After two days of incubation, the selection of cells with optimal gene editing efficiency was carried out using the GeneArt Genomic Cleavage Detection Kit (Thermo Fisher Scientific). The equation to calculate the cleavage efficiency and the list of primers for the cleavage assay are indicated in Supplementary Table S1. Knockdown of YKL-40 was confirmed by qRT-PCR and Western blot analyses. To prove that the phenotypic changes seen in the knockdown cells were specifically due to the lack of YKL-40 rather than to off-target effects of the CRISPR/Cas9 system or to stress responses, YKL-40.KD cells were transfected with a YKL-40 “rescue” expression plasmid, pCMV3-YKL-40-GFPSpark (Sino Biological). This plasmid was also used to obtain YKL-40.OE cells by transfecting cells using the JetOPTIMUS^®^ DNA Transfection Reagent (Polyplus-transfection S.A.) according to the manufacturer’s instructions. Cells transfected with the empty pCMV vector were considered the negative control. Two days after transfection, fluorescent cells were observed at 20× magnification. Bright-field images and fluorescence images using DAPI and GFP filter sets (DAPI excitation 361 nm, emission 486 nm; GFP excitation 488 nm, emission 510 nm) were acquired. All images were taken separately and analyzed to assess the percentage of transfected cells. A yield of transfection >80% was considered significant. Cells were harvested 48 h post-transfection, lysed for RNA, and used in different experimental procedures.

### 2.3. Western Blot Analysis

Total protein extracts were obtained from lysed cells using RIPA buffer plus proteinase inhibitors (Sigma-Aldrich, Steinheim, Germany). Proteins (20 µg) were submitted to Bolt 10% Bis-Tris Plus gel (Thermo Fisher Scientific). Then, separated proteins were electroblotted onto a Nitrocellulose filter. The following primary antibodies were used for immunoblotting: anti-YKL-40 (AbCam, Cambridge, UK, #ab180569, 1:300) and anti-β-Actin (Santa Cruz Biotechnology, Santa Cruz, CA, USA, #sc-47778, 1:1000). Bound primary antibodies were visualized using Clarity Max Western ECL Substrate (Bio-Rad, Pleasanton, CA, USA) on a Chemidoc Imaging System (Bio-Rad, Pleasanton, CA, USA). Each experiment was repeated three times. Band intensity was quantified using Image J software and normalized to β-Actin.

### 2.4. Cell Migration, Invasion, and Proliferation Assays

Cell migration was evaluated by the wound-healing method. After 24 h transfection, cell monolayers were scraped, and images of the wounded area were captured at 0, 24, and 48 h by an inverted microscope (2× magnification). Images were analyzed using ImageJ software, and the change in the wound area over time was expressed as a percentage of wound closure. Pictures represent one of three independent experiments.

The Matrigel-based invasion assay was performed in Boyden chambers containing an 8 μm polycarbonate membrane coated with Matrigel (Corning Matrigel GFR Matrix, 2 mg/mL). Two days after transfection, 104 cells were added in suspension to the upper side of the Boyden chamber, while the bottom well contained 10% serum in the culture medium. After 24 h, migrated cells were fixed and stained with the DiffQuik Stain Kit (Baxter Diagnostics), quantified by analyzing different microscopic fields (10× magnification), and expressed as mean numbers of migrated cells. Data represent three independent experiments.

Two days after seeding, cell proliferation was assessed using the Resazurin Assay Kit (Immunological Sciences, Rome, Italy) as previously described [41]. The relative cell number was calculated from standard curves of resazurin fluorescence. Data represent three independent experiments, performed in triplicate.

### 2.5. Mice and Tissue Sample Processing

Six-week-old BALB/c female mice were used to reproduce the azoxymethane (AOM)/dextran sodium sulfate (DSS) model of CRC. The study was approved by the Italian Ministry of Health according to decree no. 336/2013-B. All animal procedures followed the institutional guidelines for laboratory animal care and adhered to ethical standards. Mice were treated as described previously [22]. Five AOM/DSS-treated and five untreated (control) mice were sacrificed at IV, VI, and XII weeks to analyze progressive CRC stages. Five AOM-treated and five DSS-treated mice were examined as controls. The large intestine was removed, opened longitudinally, and flushed with PBS. Normal colon mucosa and tumors were excised and stored at −80 °C until RNA extraction or fixed in formalin and embedded in paraffin.

### 2.6. CRC Patient Sample Collection and Storage

Twenty-two primary tumors and paired normal mucosa (*n* = 22, aged 73.77 ± 7.75) were obtained from the University Hospital Eurohospital, Plovdiv, Bulgaria, after authorization by the Ethics Committee (Protocol N. R-1838/15-07-2013). Additionally, newly diagnosed CRC patients (*n* = 19, aged 68.05 ± 10.09) between 2020 and 2021 (Protocol N: 6/20.12.2018) were included in the present study. Colon mucosa was surgically excised from each patient after signed informed consent. The specimens were quickly frozen at −80 °C. The pathomorphological assessment was performed by two independent pathologists. None of the patients underwent radiotherapy, chemotherapy, or targeted therapy prior to surgery. Venous blood samples from 43 patients and 43 healthy persons, age and gender-matched, were collected before surgery. The serum was aliquoted and stored at −70 °C before analysis. Clinical data were collected by reviewing patients’ charts (Supplementary Table S2).

### 2.7. Immunohistochemical Detection

Formalin-fixed paraffin-embedded (FFPE) sections were prepared using standard procedures for histopathological examination and immunohistochemistry (IHC). Murine tumors were diagnosed according to the histopathological criteria described by Boivin and colleagues [42]. IHC was performed on murine and human FFPE sections after antigen retrieval with sodium citrate. Anti-YKL-40/CHI3L1 antibody (Abcam, Cambridge, UK, #ab180569, 1:100) was used as a primary antibody. Nuclei were counterstained with hematoxylin. Images were observed under a microscope and analyzed using NIS FreeWare 2.10 software (Nikon, Minato, Tokyo, Japan). The image data were analyzed using ImageJ software [43]. Three to five same-sized areas per sample were chosen. The % of the stained area was quantified after adjusting the color threshold.

### 2.8. ELISA

Serum YKL-40 concentrations of both CRC patients and controls were determined by ELISA using a commercial kit (Quidel Corporation, San Diego, CA, USA) according to the manufacturer’s protocol. The validation of the method was performed in compliance with the international standard based upon BDS/EN/ISO 15189. The detection limit was 10 μg/mL. All samples were analyzed in duplicates. YKL-40 levels were measured as absorbance at 405 nm on the ELISA Sunrise Reader (Tecan, Mannedorf, Switzerland).

### 2.9. RNA Extraction and Quantitative Real-Time PCR (qRT-PCR)

Total RNA was extracted from tissues and cells using TRIzol (Invitrogen, Carlsbad, CA, USA). DNA contamination was removed by the DNA-free DNA Removal Kit (Ambion, Austin, TX, USA), and then RNA concentration and purity were assessed spectrophotometrically. YKL-40 gene expression in tissue samples was assessed using the RevertAidTM First Strand cDNA Synthesis Kit (Thermo Fisher Scientific) to obtain cDNA. qRT-PCR analysis was performed using the GreenMasterMix (Genaxxon Bioscience, Ulm, Germany) according to the manufacturer’s instructions. GAPDH, ACTINB, and UBC were used as YKL-40 normalizers. The primer sequences are listed in Supplementary Table S3A. YKL-40 and EMT gene expression in CRC cells was assessed using the iScript Advanced cDNA Synthesis Kit (Bio-Rad) to obtain cDNA. TaqMan Gene Expression Master Mix and real-time PCR assays (Thermo Fisher Scientific) were utilized for the genes listed in Supplementary Table S4. GAPDH and HPRT1 were used as normalizers. Relative gene expression was calculated according to the method of Fold Change [2^-(ΔΔCt)]. Amplifications were performed in triplicate wells.

### 2.10. CRC Patient Databases and Bioinformatics Analysis

The analysis of the YKL-40 gene was carried out in a multi-cohort study of public human CRC (hCRC) expression profiles and clinical data (*n* = 1533) based on the Affymetrix Human Genome U133 Plus 2.0 microarray platform. Six cohorts (Supplementary Table S4) were collected from the Gene Expression Omnibus (GEO) repository and The Cancer Genome Atlas (TCGA-COAD) using the GEOquery R package [44,45]. Cohort 1 (stage I–III CRC patients (*n* = 226, GSE14333)) [46] and cohort 2 (stage II–III CRC patients (*n* = 130, GSE37892)) [47] were used to investigate the disease-free survival (DFS), meaning the difference between the time of surgery and the time of death or of cancer recurrence. Cohort 3 (stage I–III CRC patients (*n* = 125, GSE41258)) [48] allowed the calculation of the cancer-specific survival (CSS), considering death events only if related to cancer. Cohort 4: data of I-IV CRC patients (*n* = 585, GSE40967), with or without KRAS mutation [49], were used to examine the relapse-free survival (RFS), defined as the length of time after primary treatment that the patient survives without any signs or cancer symptoms. Cohort 5: data of mCRC patients (*n* = 80, GSE5851), treated with cetuximab monotherapy, with or without KRAS mutation [50], were used to compute the progression-free survival (PFS), defined as the time from study enrollment to disease progression or death. Cohort 6: data of stage I–IV CRC patients (*n* = 387, TCGA) [51] were used to calculate the Overall Survival (OS), i.e., the time from study enrollment to death. Patients having mucinous adenocarcinoma were excluded. Furthermore, data of patients that received oxaliplatin monotherapy (*n* = 88) were available.

### 2.11. Statistical Analysis 

GraphPad Prism software was used for the graphical representation of data. Statistical analyses of qRT-PCR experiments and in vitro assays were performed using two-sided Student’s *t* test. Fisher’s exact test was applied to determine the presence of nonrandom associations between clinical characteristics. In all statistical analyses, *p* < 0.05 was considered statistically significant. All data are shown as the mean ± standard deviation.

The analysis of gene expression data and other statistical analyses on public datasets were performed in R ver. 3.6.0 (http://www.r-project.org). Patients were dichotomized through the Maximally Selected Rank Statistics (maxstat) R package to determine the optimal cutpoints of continuous variables (i.e., gene expression values), which best split individuals into high and low YKL-40. Prognostic significance was estimated by log-rank tests and plotted as Kaplan–Meier curves. Multivariate Cox proportional hazards regression analysis was used to evaluate the effect of clinical, genetic, and treatment variables when one of the following events happened: OS, DFS, PFS, RFS, and CSS.

The discriminatory power of YKL-40 in tissue and serum samples was assessed by estimating the area under the receiver operating characteristic (ROC) curves using molecular and protein expression levels in cancer-free subjects and CRC patients. The area under the curve (AUC)’s 95% confidence intervals of the sensitivity at given specificity points were computed with 2000 stratified bootstrap replicates. The optimal cutoff point (more than one if on an equal footing) corresponded to the maximum Youden’s index from the ROC curve.

## 3. Results

### 3.1. Generation of Genetically Engineered CRC Cells for YKL-40 Expression

**Figure 1:**
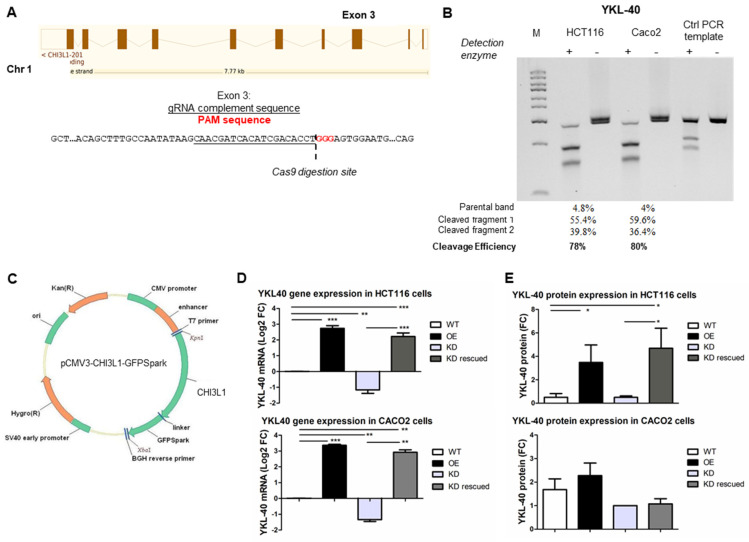
Knockdown and re-/overexpression of YKL-40 in HCT116 and Caco2 cells. (**A**) Knockdown via CRISPR/Cas9 approach is described. Human YKL-40 is located on chromosome 1. The exon 3 sequence is reported, the gRNA complement sequence is underlined, and the PAM sequence is indicated with red nucleotides. (**B**) Gel image of Genomic Cleavage Detection Assay using DNA extracted from transfected HCT116 and Caco2 cells. Control template and primers were included as a PCR technical control. Cleavage efficiency is indicated for each sample. (**C**) Map of pCMV-CHI3L1-GFPSpark plasmid used for YKL-40 gene expression. Validation of YKL-40 knockdown and re-/overexpression at (**D**) mRNA and (**E**) protein levels in both cell lines. qPCR data and densitometric data are shown as mean ± SD. Statistically significant differences were calculated using Student’s *t* test: *, *p* < 0.001; **, *p* < 0.01; ***, *p* < 0.0001. Data represent three independent experiments.

To explore the role of YKL-40 in CRC growth and progression, two hCRC cell lines with different KRAS and p53 mutational status, HCT116 and Caco2, were subjected to YKL-40 knockdown (YKL-40.KD), re-expression (YKL-40.KD rescued), and overexpression (YKL-40.OE). Technical control groups (cells treated with transfection reagents only) gave similar results to untreated controls (YKL-40.WT) in all experiments (Supplementary Figure S1). For YKL-40 silencing, we successfully targeted exon 3 of the CHI3L1 gene using the CRISPR/Cas9 system described in Figure 1A, Supplementary Figure S1. We achieved a high gene editing efficiency in both HCT116 (78%) and Caco2 (80%) cells (Figure 1B). Similarly, elevated transfection efficiency (>80%) was obtained in YKL-40.OE cells transfected with pCMV3-YKL-40-GFPSpark plasmid (Figure 1C), as shown by fluorescence microscopy (Supplementary Figure S1). For further validation, YKL-40 silencing/overexpression in both cell lines was confirmed by qRT-PCR (Figure 1D) and Western blotting (Figure 1E, Supplementary Figure S1).

### 3.2. YKL-40 Affects the Migratory Potential, Invasion, and Proliferation of CRC Cells

**Figure 2:**
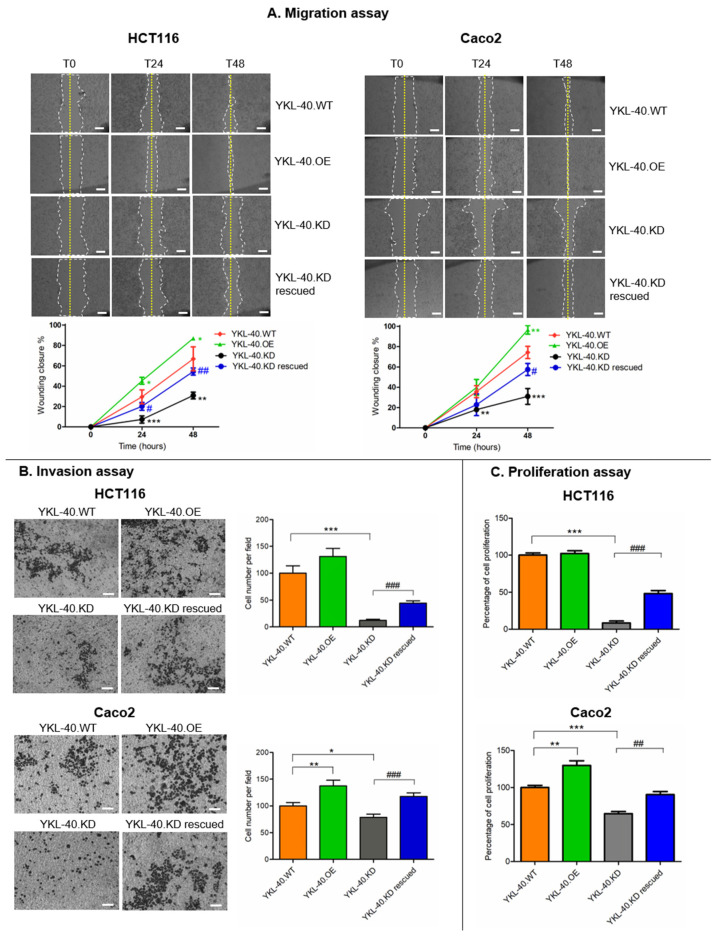
YKL-40 expression correlates with migration (**A**), invasion (**B**), and proliferation (**C**) in HCT116 and Caco2 colon cancer cell lines. Representative images of migratory (2× magnification; scale bar 500 µm) and invasive cells (10× magnification; scale bar 50 µm) are shown. Cell migration is expressed as wounding closure percentage at 0 h (T0), 24 h (T24), and 48 h (T48) after wounding. For both migration and invasion assays, pictures represent one of three independent experiments. Cell proliferation of HCT116 and Caco2 cell lines following YKL-40 gene expression modulation was assessed based on the results of the Resazurin assay. Data represent three independent experiments, performed in triplicate. Data are shown as mean ± SD. Statistically significant differences were calculated using Student’s *t* test: *, normalization vs. YKL-40.WT; *, *p* < 0.01; **, *p* < 0.001; ***, *p* < 0.0001; #, normalization vs. YKL-40.KD; #, *p* < 0.01; ##, *p* < 0.001; ###, *p* < 0.0001.

We investigated whether YKL-40 plays a functional role in the CRC metastatic phenotype. As shown in Figure 2A, the wound-healing assay revealed the effect of YKL-40 on the migratory potential of HCT116 and Caco2 cells. In fact, YKL-40.OE cells significantly enhanced their ability to migrate into the wounded area compared to control cells. By contrast, endogenous YKL-40.KD led to a significant reduction in wound closure. In both cell lines, the effect of YKL-40.KD on the migration rate was more pronounced at 48 h compared to 24 h, and this effect was partially rescued after YKL-40 re-expression.

Consistent with the cell migration data, YKL-40.OE cells displayed increased invasion through Matrigel in comparison with control cells, while the suppression of the YKL-40 gene in both cell lines reduced the number of invading cells. This effect was partially recovered following gene re-expression (Figure 2B).

The modification of YKL-40 gene expression also impacted cell proliferation (Figure 2C). YKL-40.OE Caco2 cells showed a significantly increased proliferation rate, more markedly than HCT116 cells, whereas both YKL-40.KD cell lines showed reduced proliferation that was rescued after YKL-40 gene re-expression.

### 3.3. YKL-40 Promotes Cell Migration/Invasion via Regulating EMT-Related Genes in CRC Cells

**Figure 3:**
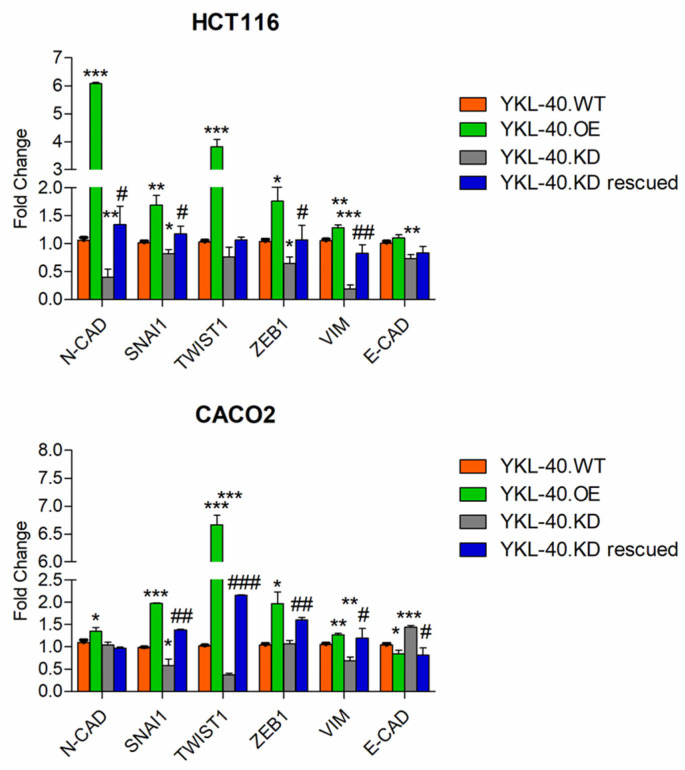
EMT marker expression regulation in HCT116 and Caco2 cells with modulated YKL-40 gene expression. qPCR data are shown as mean ± SD. Statistically significant differences were calculated using Student’s *t* test: *, normalization vs. YKL-40.WT; *, *p* < 0.01; **, *p* < 0.001; ***, *p* < 0.0001; #, normalization vs. YKL-40.KD; #, *p* < 0.01; ##, *p* < 0.001; ###, *p* < 0.0001. Data represent three independent experiments.

To reveal a correlation between YKL-40 and the EMT mechanism in promoting CRC cell migration and invasion, we analyzed the expression levels of different EMT-associated genes in genetically engineered CRC cells (Figure 3). qRT-PCR results showed that both mesenchymal markers (N-cadherin and Vimentin) and EMT activators (Twist1, Snai1, and Zeb1) were upregulated in YKL-40.OE cells and downregulated in YKL-40.KD cells compared to YKL-40.WT cells. Consistent with this, the majority of EMT makers were re-expressed in YKL-40.KD rescued cells. A particular gene expression pattern was observed for N-cadherin and E-cadherin in both cell lines. In fact, N-cadherin levels were more affected by YKL-40 genetic manipulation in HCT116 than in Caco2 cells.

### 3.4. YKL-40 Is Highly Expressed from ACF to Adenocarcinoma Stages in a CRC Murine Model

**Figure 4:**
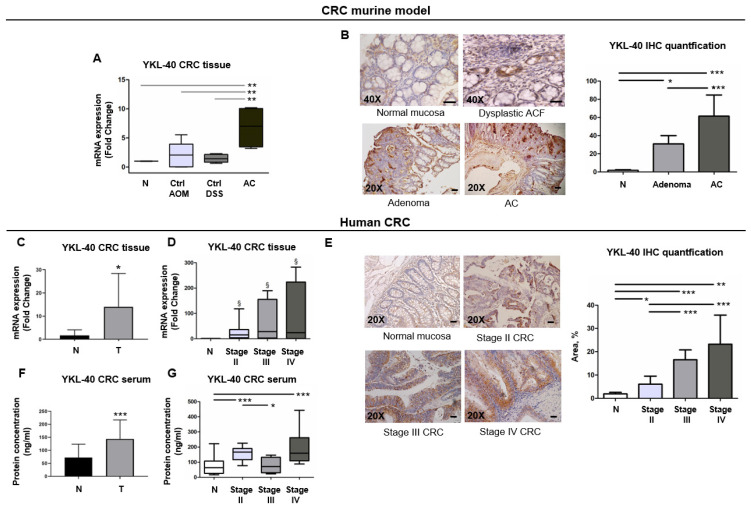
YKL-40 is overexpressed in CRC mouse model and can be used for differentiation of cancerous from normal mucosa. (**A**) qPCR analysis of YKL-40 mRNA levels in murine colon normal mucosa (N) (*n* = 5) and only-AOM- (Ctrl AOM) (*n* = 5) or only-DSS-treated (Ctrl DSS) (*n* = 5) mucosa and adenocarcinoma (AC) (*n* = 5). (**B**) IHC analysis of YKL-40 protein in murine dysplastic ACF, adenoma, and adenocarcinoma (AC) and its quantification. (**C**) qPCR analysis of YKL-40 mRNA levels in tumoral and matched non-tumoral tissues of CRC patient samples (*n* = 41) and (**D**) in samples stratified for CRC stages. (**E**) IHC analysis of YKL-40 protein in human colorectal tumoral and normal tissues and its quantification. (**F**) Measurement of YKL-40 protein levels both in normal individuals’ (*n* = 43) and in CRC patients’ (*n* = 43) sera and (**G**) in samples stratified for CRC stages. (**A**–**G**) Data are shown as mean ± SD. Statistically significant differences were calculated using Student’s *t* test: *** *p* < 0.0001; ** *p* < 0.001; * *p* < 0.01; § *p* = 0.1. (**B**,**E**) 20× and 40× magnification. Scale bars, 50 µm.

Based on the above results, we hypothesized that YKL-40 expression might be higher in colorectal tumors with more advanced malignancy. Therefore, we analyzed YKL-40 expression in the AOM/DSS murine model, because we previously verified its ability to recapitulate the multiphase colorectal carcinogenesis observed in hCRC [22]. As expected, AOM/DSS mice sacrificed at 4 weeks presented the earliest histopathological manifestations of colon lesions, aberrant crypt foci (ACF), and dysplastic microadenomas. Adenomas and polypoid tumors were detected at 6 and 12 weeks, respectively (Figure 4A). Some of the adenocarcinomas also displayed the ability to invade the mucosa. At 12 weeks, mice that received only AOM or only DSS showed histologically normal colons (Supplementary Figure S2). In the AOM/DSS model, we found that YKL-40 was overexpressed in adenocarcinomas compared to control colon mucosa, as validated by IHC (Figure 4A,B). In addition, IHC analysis showed larger stained areas in adenocarcinomas compared to adenomas and detectable YKL-40-positive staining in dysplastic ACF (Figure 4B).

### 3.5. YKL-40 Is Overexpressed in Cancer Tissues and Sera of Patients with mCRC and Has the Potential to Discriminate CRC Cases from Controls

We validated the preclinical results in hCRC tissues by using a training set (*n* = 19) of patients with different invasiveness (Supplementary Table S2). The transcriptional level of YKL-40 was significantly upregulated in tumors compared to normal mucosa, as confirmed by IHC (Figure 4C–E). In addition, we found an upward trend in YKL-40 expression associated with the more advanced stages (Figure 4D,E). Furthermore, the mean serum value of YKL-40 in CRC patients was significantly higher than in healthy subjects (Figure 4F). Interestingly, although an increasing trend in the serum YKL-40 concentration associated with the more advanced stages was not found, stage IV CRC patients showed the highest circulating level of the protein (Figure 4G).

**Figure 5:**
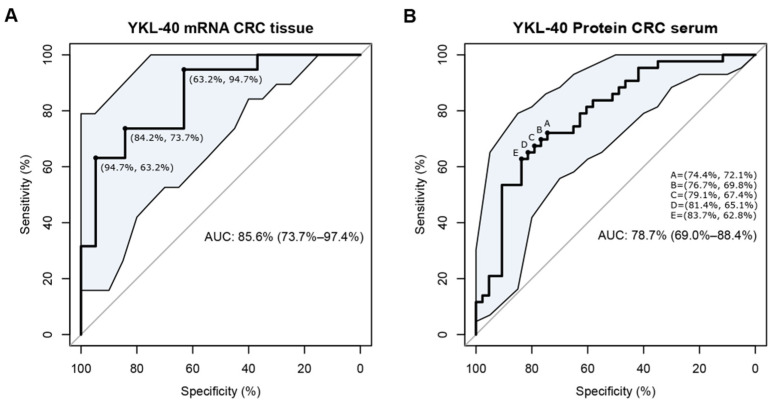
Diagnostic performance of YKL-40 mRNA levels in CRC tissues (H) and YKL-40 protein levels in CRC serum. Receiver operating characteristic (ROC) curve analysis in (**A**) CRC tissue samples (*n* = 19) and colon mucosa derived from cancer-free subjects (*n* = 19); area under the curve (AUC) = 85.6 (95% CI: 73.7–97.4%, *p* = 0.0002); (**B**) tumor serum samples (*n* = 43) and serum samples derived from cancer-free subjects (*n* = 43); area under the curve (AUC) = 78.7 (95% CI: 69.0–88.4%, *p* = 0.000004).

To determine whether the high CRC tissue and serum levels of YKL-40 can be used as a potential biomarker for distinguishing CRC patients from healthy controls, ROC analysis was performed. A ROC curve was drawn to assess the discriminatory power of the YKL-40 expression levels in CRC tissue samples and colon mucosa derived from cancer-free subjects (Figure 5A). The AUC value was 85.6 (95% CI: 73.7–97.4%, *p* = 0.0002). ROC curve analysis and the Youden Index identified an optimal cutoff of 0.58. Similarly, a ROC curve analysis was performed for tumor serum samples and serum samples derived from cancer-free subjects. The AUC value was 78.7 (95% CI: 69.0–88.4%, *p* = 0.000004). According to this ROC curve analysis, the optimal cutoff was 0.47 (Figure 5B). Taken together, these findings demonstrate that both transcription and protein YKL-40 levels have great potential as diagnostic biomarkers in CRC.

Further validation was performed on a larger number of patients included in six cohorts of a public microarray dataset. We found that patients had a higher expression of the YKL-40 gene in four out six cohorts (Supplementary Table S3B). Moreover, although not significant, YKL-40_high_ patients apparently had more advanced disease than YKL-40_low_ patients in cohorts 1, 2, and 3, while patients with YKL-40_high_ expression were much more abundant in cohort 5, which consisted only of patients with stage IV CRC (Supplementary Table S5).

### 3.6. YKL-40 Shows Prognostic Significance in Patients with mCRC

**Figure 6:**
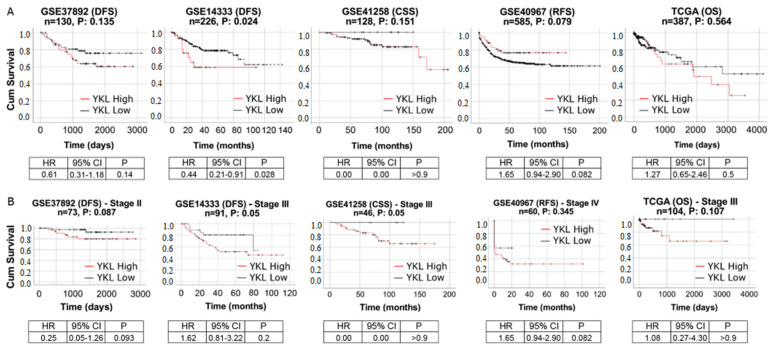
Kaplan–Meier survival curves of high YKL-40 (dashed line) versus low YKL-40 (solid line) for (**A**) all patients of cohorts 1–4 and cohort 6 and for (**B**) patients with advanced CRC stages from the same cohorts. Expression value thresholds for determining high and low groups were determined through maxstat R package, except for the analysis of all patients of TCGA, where it was determined with YKL-40 median expression threshold. *p*-values were calculated using log-rank tests. Tick marks represent censored data.

We investigated the prognostic impact of YKL-40 by analyzing data of CRC patients with different stages. In four out five datasets, Kaplan–Meier curves showed shorter survival in YKL-40_high_ patients than in YKL-40_low_ patients (Figure 6A), indicating that YKL-40 upregulation is related to a poor prognosis of CRC. This was emphasized in each cohort stratified for YKL-40 expression and in stage II, III, and IV CRC patients considered separately. In particular, a significant negative prognostic role was observed in YKL-40_high_ patients with stage III CRC (Figure 6B). For stage II and stage IV CRC, *p*-values were not remarkably far from the standard 0.05 threshold, indicating a clear trend of significance.

Afterwards, we conducted univariate and multivariate analyses of factors affecting the survival rates to determine whether the prognostic impact of the YKL-40 gene expression pattern is independent of other clinical variables. To reduce erroneous outcomes due to lower dataset dimensionality, we analyzed Cohort 1-GSE14333 (*n* = 226, CRC stages I, II, and III) and Cohort 4-GSE40967 (*n* = 585, CRC stages I, II, III, and IV). When highly expressed in both the first dataset (*p*-value = 0.0172) and the second dataset (*p*-value = 0.0918), YKL-40 was found to be a negative prognostic variable since the YKL-40_low_ status was related to better DFS rates than YKL-40_high_. The multivariate analysis showed that this was independent of other clinical variables (Supplementary Table S6).

### 3.7. YKL-40 Shows Predictive Significance in mCRC Patients Treated with Cetuximab and Oxaliplatin

**Figure 7:**
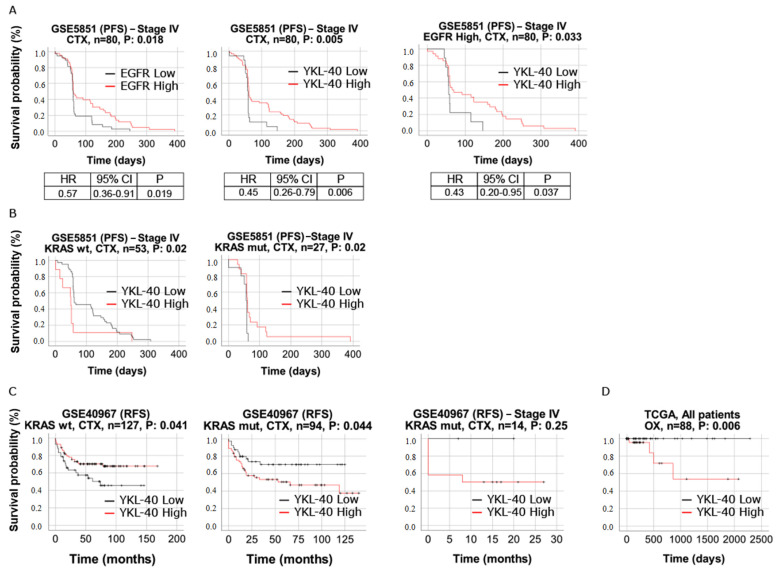
(**A**) Kaplan–Meier survival curves of YKL-40_high_ (solid line) versus YKL-40_low_ (dashed line) and EGFR_high_ (solid line) versus EGFR_low_ (dashed line) patients of cohort 5 (GSE5851). Survival curves of YKL-40_high_ (solid line) versus YKL-40_low_ (dashed line) patients belonging to the EGFR_high_ group. (**B**) Survival curves of YKL-40 for patients of cohort 5 with WT-KRAS and mutant KRAS. (**C**) Survival curves of YKL-40 for patients of cohort 4 (GSE40967) with WT-KRAS and mutant KRAS. (**D**) Survival curves of YKL-40_high_ (solid line) versus YKL-40_low_ (dashed line) patients of cohort 6 (TCGA-COAD). For all survival curves, *p*-values were calculated using log-rank tests, and expression value thresholds for determining high and low groups were determined through maxstat R package. Abbreviations, CTX, cetuximab; OX, oxaliplatin.

We evaluated the response to cetuximab in the 80 mCRC patients of cohort 5 treated with cetuximab monotherapy. Significantly longer survival (PFS), i.e., a better response to therapy, was associated with elevated EGFR gene expression. The same was observed in YKL-40_high_ patients (Figure 7A), in line with the study by Liu and colleagues showing an improvement in sensitivity to cetuximab mediated by YKL-40 [52]. Interestingly, we found statistically significant differences in the response to cetuximab in mCRC patients with EFGR_high_ stratified for YKL-40 expression. In particular, PFS was longer in patients with the upregulation of both YKL-40 and EGFR, and the HR was slightly lower than only-EFGR_high_ and only-YKL-40_high_ patients (Figure 7A), suggesting that YKL-40_high_ patients with EGFR_high_ may die at a lower rate per month than patients with only EGFR_high_ or only YKL-40_high_. Moreover, the multivariate analysis showed that in cohort 5, the prognostic relevance of the YKL-40 gene is independent of classic clinical prognostic features (Supplementary Table S7).

Information on the KRAS mutation status was also provided for patients in cohorts 4 and 5. Although strictly not significant, there was a difference in the KRAS mutation rates between the YKL-40_high_ and YKL-40_low_ expression groups in both cohorts (Supplementary Table S5). Interestingly, in cohort 5, a higher percentage of WT-KRAS in YKL-40_high_ patients than in YKL-40_low_ patients was observed, but the Kaplan–Meier curves for the response to cetuximab revealed no differences between YKL-40_high_ and YKL-40_low_ patients with WT-KRAS (Figure 7B). On the contrary, KRAS-mutant patients with YKL-40_high_ showed a significantly longer PFS duration than KRAS-mutant patients with YKL-40_low_ (Figure 7B), suggesting that the role of YKL-40 in the resistance to cetuximab treatment could surprisingly counteract the effect of KRAS mutation on the response to cetuximab. Cohort 4 allowed the evaluation of YKL-40’s prognostic role in combination with KRAS mutation in CRC patients not treated with cetuximab. In this case, a poor prognosis was observed in patients who were KRAS-mutant and YKL-40_high_. This trend was confirmed in stage IV CRC, although it did not reach significance (Figure 7C).

Finally, we evaluated the role of the YKL-40 expression level in the response to oxaliplatin therapy. In this case, significantly shorter survival (OS) was associated with elevated YKL-40 gene expression (Figure 7D).

## 4. Discussion

Over the past decade, considerable attention has been focused on the potential role of YKL-40 in the development of a variety of human cancers. The present study is the first attempt to evaluate the clinicopathological significance of YKL-40 tissue expression in mCRC.

Initially, we explored the relationship between YKL-40 and tumor progression hallmarks in CRC using KRAS-mutant HCT116 cells and p53-mutant Caco2 cells subjected to YKL-40 gene silencing and overexpression. In both cell lines, regardless of the genetic background, YKL-40 expression caused changes in CRC cell migration, invasion, and proliferation, three crucial characteristics of advanced tumors. The wound-healing assay showed that the expression level of YKL-40 is closely related to the migration rate of CRC cells, in agreement with what was observed in endothelial cells [53], glioblastoma [37], and prostate cancer cells [54]. The invasion assays revealed a determinant role of YKL-40 in promoting the invasion of CRC cells, thus implying an increase in metastatic potential, as observed in other tumors [31]. In both genetically engineered cell lines, we observed that the YKL-40 mRNA level was significantly lower after knockdown and higher after overexpression, while the effect at the protein level was consistent with the molecular results but not statistically significant, particularly in Caco2 cells. This may be due to a possible effect of KRAS mutation in HCT116 cells or p53 absence in Caco2 cells on YKL-40 protein expression, as a correlation between the expression of p53 or KRAS and YKL-40 has already been suggested [55,56]. Further YKL-40 post-translational regulation may be linked to PI3K/AKT [57] and JNK/ERK [58] pathways that play a crucial role in CRC progression [59]. However, all of these mechanisms require further investigation in the future.

Since the EMT mechanism is associated with over 90% of metastatic pathways of malignant tumors [60], we investigated whether the involvement of YKL-40 in the metastatic phenotype shown by YKL-40.OE HCT116 and Caco2 cells depends on EMT induction. Molecular analysis showed a significant association between the overexpression of YKL-40 and mesenchymal cell markers and the downmodulation of the epithelial marker E-cadherin, while YKL-40.KD cells showed the opposite molecular patterns. Interestingly, N-cadherin expression levels did not correspond to those of EMT markers in Caco2 cells. Since YKL-40 reportedly regulates p53 and E-cadherin gene expression, we may speculate the likely involvement of YKL-40 in the modulation of N-cadherin expression via p53, although more research on this mechanism is needed.

In general, our results suggest the involvement of YKL-40 in promoting the migration and invasion of CRC cells by activating the EMT mechanism, probably through the PI3K/AKT signaling pathway, which is involved in the direct induction of EMT [61], and the Ras/Raf/MEK/ERK signaling pathway [62].

Hao et al. concluded that YKL-40 promotes bladder cancer metastasis by regulating EMT genes such as E-cadherin, Twist, Snail, Slug, N-cadherin, and vimentin [63]. In our study, we also examined the same set of EMT-related markers, so we can assume that the glycoprotein plays a similar role in CRC.

A similar role of YKL-40 as a possible EMT activator has also recently been reported in prostate cancer [64], bladder cancer [63], and NSCLC [65].

It was determined that YKL-40 participates in the regulation of phosphatidylinositol 3 kinase (PI3K)/AKT/mTOR pathway or Ras/Raf/MEK/ERK cascade, which is related to tumor survival, transformation, invasion, and metastasis [66]. Synergistically, the induction of the PI3K/AKT pathway is involved in cancer migration and invasion enhancement [60]. Our study may amplify previous evidence, where YKL-40 was shown to promote the migration and invasion of cancer cells by regulating EMT genes via the AKT signaling pathway [31].

This highlights YKL-40 as a potential factor in the metastasis of these types of tumors as well as of CRC and thus as a promising therapeutic target.

Regarding cell proliferation, we found that YKL-40.KD inhibits and YKL-40.OE increases the growth of both CRC cell lines, although YKL-40.OE Caco2 cells showed a significantly higher proliferation rate than HCT116 cells. This is in line with a recent study of CRC highlighting a significant increase in the cell proliferation rate in YKL-40.OE cells associated with p53 downregulation and EGFR upregulation [52]. Although the involvement of YKL-40 in cell proliferation has been extensively investigated in physiological conditions or in various inflammation diseases [67,68], our findings provide evidence for this mechanism in CRC cells.

We further analyzed the expression profile of murine and human YKL-40 in CRC tissues. At the molecular level and immunohistochemically, we found YKL-40 overexpression in murine adenocarcinomas compared to control mucosa. Interestingly, the IHC signal was stronger in adenocarcinoma than in the adenoma stage and was already visible in dysplastic ACF, revealing early upregulation and a trend of increasing expression of the protein in multistage colorectal carcinogenesis. Preclinical data were validated in a training set of hCRC samples, which confirmed the significant upregulation of YKL-40 transcriptional levels in adenocarcinoma. We also found higher YKL-40 levels in the sera of CRC patients than in healthy subjects, in line with data presented by other studies [67,69]. Our findings underline the clinical significance of YKL-40, as it could efficiently distinguish CRC cases from controls with an AUC of 85.6 (95% CI: 73.7–97.4%, *p* = 0.0002) for YKL-40 mRNA in tissues and an AUC of 78.7 (95% CI: 69.0–88.4%, *p* = 0.000004), with cutoff values of 0.58 and 0.47, respectively. Moreover, as we analyzed samples from CRC patients free of other malignancies, we could speculate that YKL-40 expression is CRC-specific. These findings demonstrate that tissue and serum YKL-40 could be used as a biomarker for CRC, which can be validated through further studies on other patient cohorts.

Remarkably, the analysis of tissue samples showed an upward trend in YKL-40 expression associated with more advanced CRC stages. This observation was confirmed by the analysis of YKL-40 expression in cohorts from a public microarray database. We found pronounced overexpression in advanced hCRC stages of cohorts 1, 2, and 3 and also in patients with stage IV CRC belonging to cohort 5. These results are consistent with recent clinical findings in prostate cancer, where the increased expression of YKL-40 was observed in advanced tumor stages at both tissue and serum levels [70,71] and in breast cancer [72].

In serum samples, although an increasing trend in the serum YKL-40 concentration associated with the more advanced stages was not found, stage IV CRC patients showed the highest circulating level of the protein. Similarly, in our previous studies, serum YKL-40 was elevated in high-grade glioma [73] and in astrocytoma, as shown later by Urbanavičiūtė and colleagues [74]. Nevertheless, other confirmatory studies in larger and independent sample cohorts will be needed to explore the value of the serum detection of YKL-40 in progressive CRC stages.

In the public database of CRC patients, we also found an interesting correlation between YKL-40 expression and the KRAS mutation status. We indeed found that YKL-40 expression was prominently elevated on the KRAS-wild type background, in line with previous findings [56].

Since we showed that the high tissue expression of YKL-40 is associated with a more aggressive phenotype with metastatic potential in CRC, we explored the possible role of tissue YKL-40 level as a prognostic marker, as already proposed for circulating YKL-40 [75]. A key finding of our study is that YKL-40 expression had a reliable prognostic and predictive significance when evaluated in a large number of CRC patients considering different clinical endpoints (OS, DFS, CSS, and PFS). The analysis of the public CRC datasets revealed that the majority of the patients had a high expression of the YKL-40 gene. Moreover, on the basis of the available clinical outcome data, we observed much shorter survival in YKL-40_high_ patients than in YKL-40_low_ patients, indicating that YKL-40 upregulation correlates with a poor prognosis of CRC. Interestingly, the poor survival of CRC patients observed in all cohorts was confirmed in each cohort stratified for YKL-40 expression in a single tumor stage at a time. These results are the first to suggest a prognostic role of tissue YKL-40 in patients with advanced CRC. The univariate and multivariate analyses also indicated that the prognostic relevance of YKL-40 in CRC patients is maintained even when taking into account classical clinical prognostic features.

Resistance to therapy remains one of the most critical issues in treating CRC. For example, up to 60% of patients with WT-KRAS tumors do not respond to cetuximab therapy (5). Since the functions of YKL-40 in the sensitivity to cetuximab or other drugs are poorly explored, we investigated the possible predictive role of tissue YKL-40 by analyzing cohorts 4, 5, and 6, i.e., patients treated with cetuximab and oxaliplatin. With regard to cetuximab, we explored YKL-40-stratified patients according to the therapy response, EGFR expression level, and KRAS mutation status. Remarkably, in cohort 5, increased EGFR gene expression was significantly associated with better PFS for all patients, consistent with the role of EGFR as a target of cetuximab [76]. Surprisingly, YKL-40_high_ patients displayed a similar correlation with the clinical outcome (PFS), in line with recent data demonstrating the ability of YKL-40 to increase the sensitivity to this drug [52]. Moreover, patients with high expression levels of both EGFR and YKL-40 survived at a higher rate per month than patients with only EGFR_high_, suggesting a possible additive effect of YKL-40_high_ and EGFR_high_ expression on the response to cetuximab. The multivariate analysis showed that the prognostic relevance of the YKL-40 gene in patients with mCRC treated with cetuximab is independent of other clinical prognostic features. Furthermore, in patients of cohort 5 with mutant KRAS, the YKL-40_high_ expression status was significantly associated with a longer PFS duration, i.e., a better response to cetuximab treatment. This suggests that YKL-40 overexpression may have a role in the resistance to cetuximab treatment and could somehow improve the drug response in the subgroup of KRASmut patients. This could lead to evaluating the possible role of YKL-40 as a useful marker for selecting patients for cetuximab therapy in the setting of patients with K-ras mutations.

In addition to the role of the KRAS mutation status as a biomarker for anti-EGFR therapy in CRC patients, we also considered it as a possible prognostic factor in combination with YKL-40 expression. In cohort 4, we observed a poor prognosis in those patients who were KRAS-mutant and YKL-40_high_, a trend confirmed in stage IV CRC patients, although not statistically significant. This could lead to speculating on a cumulative effect of YKL-40 upregulation and KRAS dysregulation, which may converge on the aberrant regulation of RAS/RAF/MAPK and PI3K/AKT pathways, both under the control of KRAS and YKL-40 [77]. As an effect, this could promote proliferation-related pathways and cancer development.

Finally, since mCRC patients with WT-KRAS status can benefit from EGFR inhibition-targeted therapy combined with some oxaliplatin-based regimes, we also evaluated the role of tissue YKL-40 on the response to oxaliplatin therapy. In this case, significantly shorter survival was associated with the YKL-40_high_ level, consistent with the results related to plasma YKL-40, which was proposed as an independent prognostic biomarker in patients with mCRC treated with first-line oxaliplatin-based therapy, with or without cetuximab [69]. Even though the predictive value of tissue YKL-40 levels should be further clarified by functional studies and larger in vivo investigations, our study reveals that they could be helpful in stratifying patients with CRC with a poor prognosis and cetuximab and/or oxaliplatin resistance.

## 5. Conclusions

In conclusion, our observations provide new insight into the role of YKL-40 in promoting the metastatic phenotype during colorectal carcinogenesis and offer a new rationale for the clinical use of tissue YKL-40.

## Supplementary Materials

The following supporting information can be downloaded at: https://www.mdpi.com/article/10.3390/cells11223568/s1. Figure S1: Gene editing in HCT116 and Caco2 cells; Figure S2: Macroscopic observation of the distal regions of colons and hematoxylin/eosin staining of tumors and normal colons; Table S1: List of primer sequences used for the cleavage assay; Table S2: Clinical data; Table S3: List of primer sequences used for qRT-PCR and TaqMan assays ID; Table S4: Patient characteristics in 6 cohorts analyzed; Table S5: Clinical characteristics of YKL-40_high_ and YKL-40_low_ patients in cohorts 1 to 6; Table S6: Univariate and multivariate analyses of factors affecting DFS in stage I–III patients (cohort 1) and RFS in stage I-IV patients (cohort 4); Table S7: Univariate and multivariate analyses of factors affecting PFS in patients who received cetuximab monotherapy (cohort 5).
