# Supplementary material for: Upregulation of YKL-40 Promotes Metastatic Phenotype and Correlates with Poor Prognosis and Therapy Response in Patients with Colorectal Cancer
Source: Cells. 2022 Nov 11;11(22):3568. doi: 10.3390/cells11223568 (PMC9688424; doi:10.3390/cells11223568)
Supplement: Supplementary file 1 [file cells-11-03568-s001.zip › Supplementary Figure S1.pdf]

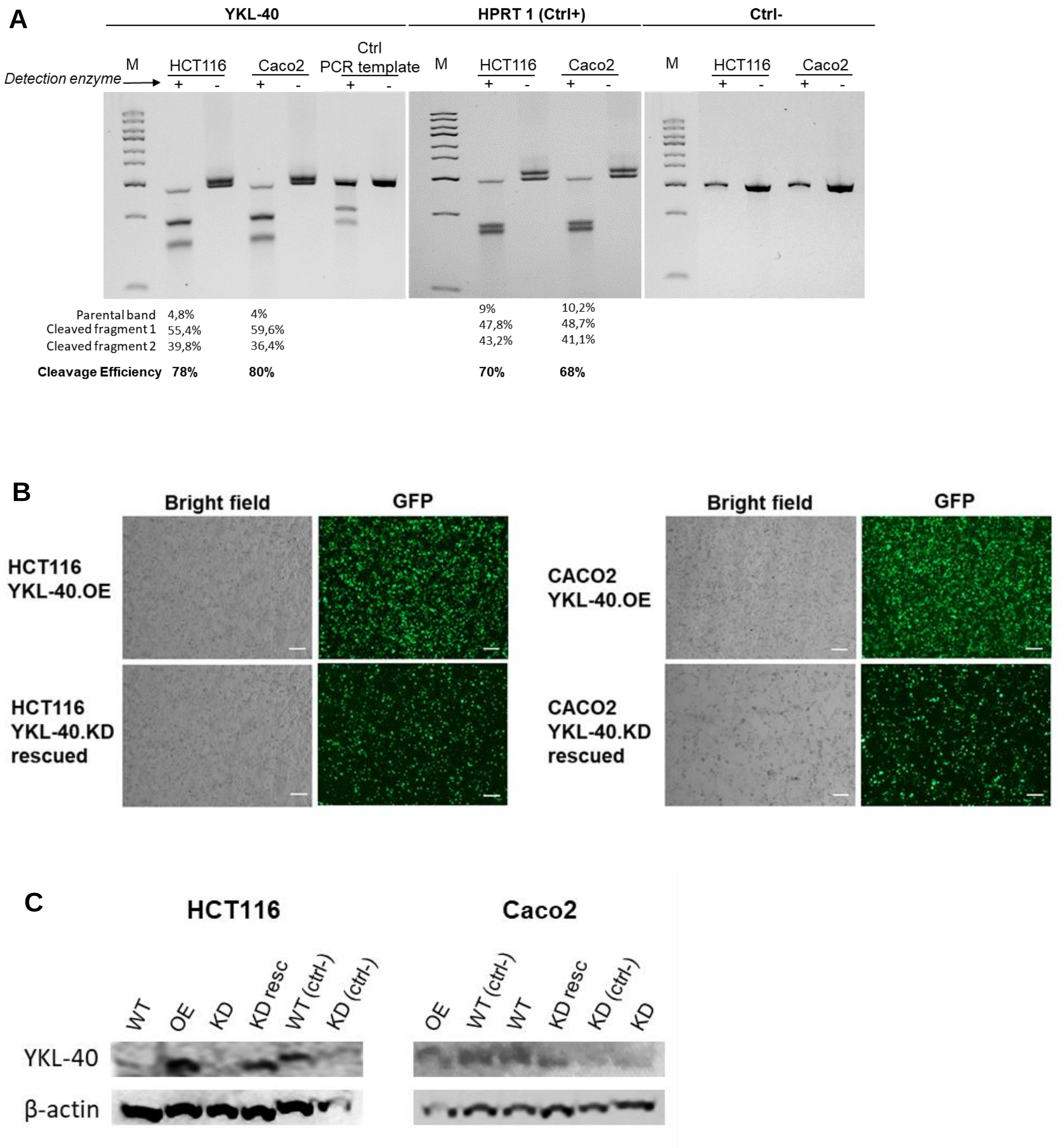

Supplementary Figure S1. A) Gel image of Genomic Cleavage Detection Assay using DNA extracted from transfected HCT116 and Caco2 cells PCR amplified using the same set of primers flanking the region of interest. After re-annealing, samples were treated with and without Detection Enzyme and run on a 2% E-Gel® EX Gel. A positive and a negative control sample for gene modification were also prepared by transfecting with TrueGuide™ gRNA Positive Control, HPRT1, and TrueGuide™ Synthetic gRNA, Negative Control. Control Template & Primers were included as PCR technical control. Cleavage efficiency is indicated for each sample. **B)** Evaluation of transfection efficiency by visualization of GFP expression in HCT116 and Caco2 cells transfected with the pCMV3-YKL-40-GFPSpark expression vector and observed under a TE200 fluorescence microscope (Nikon USA, Garden City, NY, USA). Magnification 2X, Scale bar 100 µm. **C)** Western blot analysis of YKL-40 in HCT116 and Caco2 cell lines.
