# Supplementary material for: Upregulation of YKL-40 Promotes Metastatic Phenotype and Correlates with Poor Prognosis and Therapy Response in Patients with Colorectal Cancer
Source: Cells. 2022 Nov 11;11(22):3568. doi: 10.3390/cells11223568 (PMC9688424; doi:10.3390/cells11223568)
Supplement: Supplementary file 1 [file cells-11-03568-s001.zip › Supplementary Figure S2.pdf]

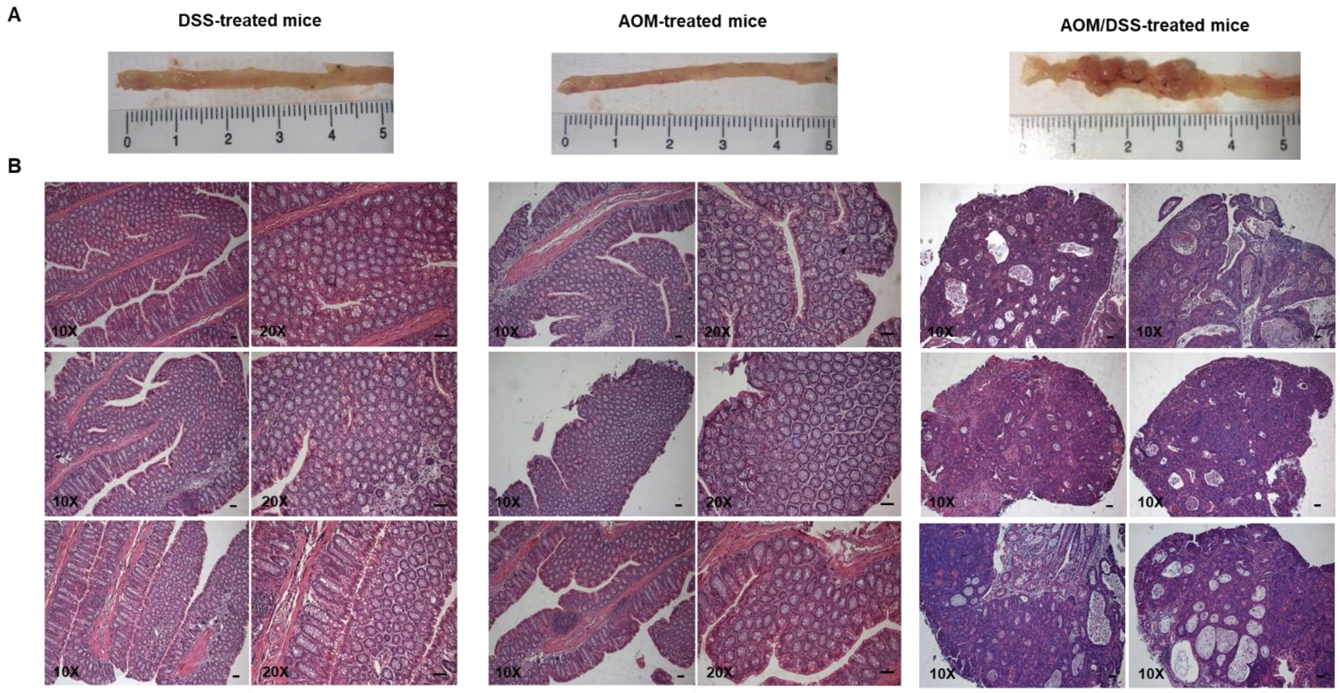

Supplementary Figure S2. A) Macroscopic observation of the distal regions of colons from DSS-, AOM- and AOM/DSS-treated mice at the end of the 12th week (1 colon per group is shown). Evident macroscopic lesions detectable only in AOM/DSS-treated colons. **B)** Hematoxylin/eosin staining of tumors and normal colons. Colon mucosae of AOM-only and DSS-only treated mice show normal histological characteristics. Adenocarcinomas with a high degree of dysplasia are detectable in AOM/DSS-treated mice. 20x original magnification. Scale bars, 50 µm.
