# Supplementary material for: Upregulation of YKL-40 Promotes Metastatic Phenotype and Correlates with Poor Prognosis and Therapy Response in Patients with Colorectal Cancer
Source: Cells. 2022 Nov 11;11(22):3568. doi: 10.3390/cells11223568 (PMC9688424; doi:10.3390/cells11223568)
Supplement: Supplementary file 1 [file cells-11-03568-s001.zip › Supplementary Table S1.pdf]

Supplementary Table S1 – List of primers sequences used for the cleavage assay.

**Gene**	**Sequence**	
CHI3L1	Fw	5’ – CTTTGCTCACAATCTGCACCC – 3’
	Rev	5’ – CCCATTTAAGCCAAGCCCCTC – 3’
HPRT1	Fw	5’ – ACATCAGCAGCTGTTCTG – 3’
	Rev	5’ – GGCTGAAAGGAGAGAACT – 3’

Equation to calculate the cleavage efficiency

Cleavage Efficiency= 1– [(1–cleaved fraction)^{½}]; Cleaved fraction= sum of cleaved band intensities/(sum of the cleaved and parental band intensities).
