# Supplementary material for: Upregulation of YKL-40 Promotes Metastatic Phenotype and Correlates with Poor Prognosis and Therapy Response in Patients with Colorectal Cancer
Source: Cells. 2022 Nov 11;11(22):3568. doi: 10.3390/cells11223568 (PMC9688424; doi:10.3390/cells11223568)
Supplement: Supplementary file 1 [file cells-11-03568-s001.zip › Supplementary Table S3.pdf]

Supplementary Table S3_A. List of primers sequences used for qRT-PCR.

Gene	Sequence	
CHI3L1	Fw	5’ – GACCACAGGCCATCACAGTCC – 3’
	Rev	5’ – TGTACCCCACAGCATAGTCAGTGT – 3’
GAPDH	Fw	5’ – AGGTCCACCACTGACACGTTG – 3’
	Rev	5’ – AGCTGAACGGGAAGCTCACT – 3’
ACTINB	Fw	5’ – AGTGTGACG TGGACATCCGGA – 3’
	Rev	5’ – GCCAGGGCAGTGATCTCCTCCT – 3’
UBC	Fw	5’ – TCCTGATCAGGCAGAGGTTGATCTT – 3’
	Rev	5’ – GACCAAGTGCAGAGTGGACTCTT – 3’

Supplementary Table S3_B – TaqMan assays ID (Applied Biosystems).

Gene	Assay ID
GAPDH	Hs99999905_m1
HPRT1	Hs01003267_m1
CHI3L1	Hs00609691_m1
CDH1 (E-CAD)	Hs01023895_m1
CDH2 (N-CAD)	Hs00983062_m1
SNAI1	Hs00195591_m1
TWIST1	Hs00361186_m1
VIM	Hs00185584_m1
ZEB1	Hs00611018_m1
