# Supplementary material for: Upregulation of YKL-40 Promotes Metastatic Phenotype and Correlates with Poor Prognosis and Therapy Response in Patients with Colorectal Cancer
Source: Cells. 2022 Nov 11;11(22):3568. doi: 10.3390/cells11223568 (PMC9688424; doi:10.3390/cells11223568)
Supplement: Supplementary file 1 [file cells-11-03568-s001.zip › Supplementary Table S4.pdf]

Supplementary Table S4 - Patient characteristics in 6 cohorts analysed.

	**Cohort 1**<br>**GSE14333 (N=226)**	**Cohort 2**<br>**GSE37892 (N=130)**	**Cohort 3**<br>**GSE41258 (N=128)**	**Cohort 4**<br>**GSE40967 (N=585)**	**Cohort 5**<br>**GSE5851 (N=80)**	**Cohort 6**<br>**TCGA (N=387)**
**Gender**						
Female	106 (46.9%)	61 (46.92%)	63 (49.6%)	263 (45%)	36 (45%)	179 (45.3%)
Male	120 (53.1%)	69 (53.08%)	65 (50.4%)	322 (55%)	44 (55%)	208 (53.7%)
**Age, years**						
Median (range)	67 (26-92)	68 (22-97)	65.7 (23-87)	69 (22-97)	60.5 (25-89)	69 (31-90)
**Location**						
Proximal	101 (44.69%)	57 (43.85%)	52 (40%)	232 (39.7%)	NA	207 (53.5%)
Distal	93 (41.5%)	72 (55.38%)	66 (52%)	351 (60%)	NA	163 (42.1%)
Rectum	30 (13.27%)	0 (0%)	10 (8%)	0 (0%)	NA	1 (0.3%)
Unknown	2 (0.88%)	1 (0.78%)	0 (0%)	2 (0.3%)	NA	16 (4.1%)
**Stage**						
**0**	0 (0%)	0 (0%)	0 (0%)	4 (0.7%)	0 (0%)	0 (0%)
I	41 (18.14%)	0 (0%)	28 (22.4%)	38 (6.5%)	0 (0%)	64 (16.5%)
II	94 (41.59%)	73 (56.15%)	51 (39.8%)	271 (46.3%)	0 (0%)	151 (39%)
III	91 (40.27%)	57 (43.85%)	49 (38.3%)	210 (35.9%)	0 (0%)	104 (26.9%)
IV	0 (0%)	0 (0%)	0 (0%)	60 (10.3%)	80 (100%)	57 (14.8%)
Unknown	0 (0%)	0 (0%)	0 (0%)	2 (0.3%)	0 (0%)	11 (2.8%)
**Adjuvant chemotherapy**						
Done	87 (38.5%)	NA	NA	240 (41%)	80 (100%)	1 (0.3%)
Undone	139 (61.5%)	NA	NA	326 (55.7%)	NA	386 (99.7%)
Unknown	0 (0%)	NA	NA	19 (3.3%)	NA	0 (0%)
**KRAS status**						
WT	NA	NA	NA	328 (56.1%)	53 (66.2%)	20 (5.2%)
Mutant	NA	NA	NA	217 (37.1%)	27 (33.8%)	15 (3.8%)
Unknown	NA	NA	NA	40 (6.8%)	0 (0%)	352 (91%)
**YKL-40 Expression**						
Low	199 (88.1%)	64 (49.2%)	14 (10.9%)	524 (89.6%)	18 (22.5%)	60 (15.5%)
High	27 (11.9%)	66 (50.8%)	114 (89.1%)	61 (10.4%)	62 (77.5%)	327 (84.5%)
