# Supplementary material for: Upregulation of YKL-40 Promotes Metastatic Phenotype and Correlates with Poor Prognosis and Therapy Response in Patients with Colorectal Cancer
Source: Cells. 2022 Nov 11;11(22):3568. doi: 10.3390/cells11223568 (PMC9688424; doi:10.3390/cells11223568)
Supplement: Supplementary file 1 [file cells-11-03568-s001.zip › Supplementary Table S5.pdf]

Supplementary Table S5 - Clinical characteristics of $YKL\text{-}40_{high}$ and $YKL\text{-}40_{low}$ patients in cohort 1 to 6.

**Cohort 1 GSE14333 (N = 226)**			
	**YKL-40 low N=199 (88.1%)**	**YKL-40 high N=27 (11.9%)**	**p-value**
**Gender[a]**			
Male	106 (53.3%)	14 (51.9%)	1
Female	93 (46.7%)	13 (48.1%)	
**Age (mean)[b]**	65.6	69.4	0.15
**Stage[a]**			
I	40 (20.1%)	1 (3.7%)	0.07
II	79 (39.7%)	15 (55.6%)	
III	80 (40.2%)	11 (40.7%)	
**Location[a]**			
Proximal R	88 (44.2%)	13 (48.2%)	0.63
Distal L	84 (42.2%)	9 (33.3%)	
Rectum	25 (12.6%)	5 (18.5%)	
Unknown	2 (1%)	0	

**Cohort 2 GSE37892 (N = 130)**			
	**YKL-40 low N=64 (49.2%)**	**YKL-40 high N=66 (50.8%)**	**p-value**
**Gender[a]**			
Male	36 (56.3%)	33 (50%)	1
Female	28 (43.7%)	33 (50%)	
**Age (mean)[b]**	69.2	67.3	0.38
**Stage[a]**			
I	0	0	0.29
II	39 (60.9%)	34 (51.5%)	
III	25 (39.1%)	32 (48.5%)	
**Location[a]**			
Proximal R	28 (43.8%)	29 (43.9%)	1
Distal L	36 (56.2%)	36 (54.6%)	
Rectum	0	0	
Unknown	0	1 (1.5%)	

**Cohort 3 GSE41258 (N=128)**			
	**YKL-40 low N=14 (10.9%)**	**YKL-40 high N=114 (89.1%)**	**p-value**
**Gender[a]**			
Male	7 (50%)	58 (50.9%)	1
Female	7 (50%)	56 (49.1%)	
**Age (mean)[b]**	57.7	65.9	0.06
**Stage[a]**			
I	4 (28.6%)	24 (21%)	0.82
II	5 (35.7%)	46 (40.4%)	
III	5 (35.7%)	44 (38.6%)	
IV	0	0	

**Location[a]**			
Proximal R	5 (35.7%)	47 (41.2%)	0.91
Distal L	8 (57.1%)	58 (50.9%)	
Rectum	1 (7.2%)	9 (7.9%)	
Unknown	0	0	
**Cohort 4 GSE40967 (N=585)**			
	**YKL-40 low N=524 (89.6%)**	**YKL-40 high N=61 (10.4%)**	**p-value**
**Gender[a]**			
Male	286 (54.6%)	36	0.58
Female	238 (45.4%)	25	
**Age (mean)[b]**	66.9	66.6	0.81
**Stage[a]**			
0	3 (0.6%)	1 (1.6%)	0.68
I	36 (6.9%)	2 (3.3%)	
II	242 (46.2%)	29 (47.5%)	
III	186 (35.5%)	24 (39.4%)	
IV	55 (10.5%)	5 (8.2%)	
Unknown	2 (0.3%)	0	
**Location[a]**			
Proximal R	211 (40.3%)	21 (34.4%)	0.53
Distal L	311 (59.3%)	40 (65.6%)	
Rectum	0	0	
Unknown	2 (0.4%)	0	
**KRAS status[a]**			
Wild type	291 (55.5%)	37 (60.7%)	0.58
Mutant	198 (37.8%)	19 (31.1%)	
Unknown	35 (6.7%)	5 (8.2%)	
**Cohort 5 GSE5851 (N = 80)**			
	**YKL-40 low N=18 (22.5%)**	**YKL-40 high N=62 (77.5%)**	**p-value**
**Gender[a]**			
Male	8 (44.4%)	36 (58%)	0.42
Female	10 (55.6%)	26 (42%)	
**Age (mean)[b]**	61.5	60.7	0.86
**KRAS status[a]**			
WT	9 (50%)	44 (71%)	0.16
Mutant	9 (50%)	18 (29%)	
Unknown	0	0	
**Cohort 6 TCGA (N=387)**			
	**YKL-40 low N=60 (15.5%)**	**YKL-40 high N=327 (84,5%)**	**p-value**
**Gender[a]**			
Male	30 (50%)	178 (54.4%)	0.57

Female	30 (50%)	149 (45.6%)	
**Age (mean)[b]**	65.2	68.4	0.09
**Stage[a]**			
I	8 (13.3%)	56 (17.1%)	0.67
II	21 (35%)	130 (39.8%)	
III	18 (30%)	86 (26.3%)	
IV	10 (16.7%)	47 (14.4%)	
Unknown	3 (5%)	8 (2.4%)	
**KRAS status[a]**			
WT	8 (13.3%)	12 (3.7%)	0.008
Mutant	3 (5%)	12 (3.7%)	
Unknown	49 (81.7%)	303 (92.6%)	

[a] χ^2-test
[b] t-test
