# Supplementary material for: Upregulation of YKL-40 Promotes Metastatic Phenotype and Correlates with Poor Prognosis and Therapy Response in Patients with Colorectal Cancer
Source: Cells. 2022 Nov 11;11(22):3568. doi: 10.3390/cells11223568 (PMC9688424; doi:10.3390/cells11223568)
Supplement: Supplementary file 1 [file cells-11-03568-s001.zip › Supplementary Table S6.pdf]

Supplementary Table S6 - Univariate and multivariate analysis of factors affecting DFS in stage I–III patients (cohort 1) and RFS in stage I-IV patients (cohort 4).

**Cohort 1 GSE14333 (N = 226)**						
	**Univariate analysis[a]**			**Multivariate analysis[b]**		
**Variables**	**N**	**5-Years DFS**	**P-value**	**HR**	**95% CI**	**P-value**
**Age**			0.2			
<70 y	125	55.3%		–	–	–
>= 70 y	101	44.7%		–	–	–
**Gender**			0.7			
Female	106	46.9%		–	–	–
Male	120	53.1%		–	–	–
**Location**			0.6			
Left	101	44.69%		–	–	–
Rectum	30	13.27%		–	–	–
Right	93	41.5%		–	–	–
Unknown	2	0.88%		–	–	–
**Adjuvant chemotherapy**			**0.02**			
Done	87	38.5%		0.79	(0.41 – 1.52)	0.48
Undone	139	61.5%		1	–	–
Unknown	0	0%				
**Stage**			**0.00005**			
I	41	18.14%		1	–	–
II	94	41.59%		3.23	(0.75 – 14.76)	0.11
III	91	40.27%		11.28	(2.52 – 50.43)	**0.0015**
IV	0	0%				
**Ykl-40 Expression**			**0.024**			
High	27	11.9%		1	–	0.0095
Low	199	88.1%		0.4	(0.19 – 0.85)	**0.0172**

[a] In univariate analyses, log-rank tests were conducted.

[b] In the multivariate Cox proportional hazard model, only variables with $P < 0.15$ in univariate analysis were included and the "enter method" was applied.

Cohort 4 GSE40967 (N = 585)						
	Univariate analysis[a]			Multivariate analysis[b]		
**Variables**	**N**	**5-Years DFS**	**P-value**	**HR**	**95% CI**	**P-value**
**Age[c]**			0.4			
<70 y	309	55.3%		–	–	–
>= 70 y	264	44.7%		–	–	–
**Gender**			**0.1**			
Female	257	46.9%		0.69	(0.51 – 0.94)	**0.02**
Male	317	53.1%		1	–	–
**Location[d]**			**0.1**			
Left	226	44.69%		1	–	–
Rectum	0	13.27%		–	–	–
Right	348	41.5%		1.27	(0.93 – 1.75)	0.132
Unknown	0	0.88%		–	–	–
**Adjuvant chemotherapy**			**<0.0001**			
Done	239	38.5%		1	–	–
Undone	319	61.5%		0.99	(0.69 – 1.43)	0.96
Unknown	16	0%		18.76	(8.62 – 40.84)	**<0.0001**
**Stage**			**<0.0001**			
I	4	0.7%		1	–	–
I	37	6.3%		11.65	–	0.99
II	267	45.6%		13.85	–	0.99
III	206	35.2%		14.52	–	0.99
IV	60	10.3%		15.55	–	0.99
**YKL-40 Expression**			**0.079**			
High	27	10.2%		1	–	0.0095
Low	199	87.9%		1.63	(0.92 – 2.87)	**0.0918**

[a] In univariate analyses, log-rank tests were conducted.

[b] In the multivariate Cox proportional hazard model, only variables with P < 0.15 in univariate analysis were included and the "enter method" was applied.

[c] Data on age of 11 patients were missing.

[d] Data on location of 11 patients were missing.
