# Supplementary material for: Upregulation of YKL-40 Promotes Metastatic Phenotype and Correlates with Poor Prognosis and Therapy Response in Patients with Colorectal Cancer
Source: Cells. 2022 Nov 11;11(22):3568. doi: 10.3390/cells11223568 (PMC9688424; doi:10.3390/cells11223568)
Supplement: Supplementary file 1 [file cells-11-03568-s001.zip › Supplementary Table S7.pdf]

**Supplementary Table S7 -** Univariate and multivariate analysis of factors affecting PFS in patients who received Cetuximab monotherapy (cohort 5).

| **Cohort 5 GSE5851 (N = 80)** | | | | | | |
|---|---|---|---|---|---|---|
| | **Univariate analysis[a]** | | | **Multivariate analysis[b]** | | |
| **Variables** | **N** | **PFS** | **P-value** | **HR** | **95% CI** | **P-value** |
| **Age[c]** | | | 0.2 | | | |
| <70 y | 54 | 67.5% | | – | – | – |
| >= 70 y | 24 | 32.5% | | – | – | – |
| **Gender**** | | | **0.009** | 1.542 | 0.9408–2.526 | 0.0858 |
| Female | 36 | 45% | | 1 | – | – |
| Male | 44 | 55% | | – | – | – |
| **YKL-40 Expression** | | | | | | |
| High | 62 | 77.5% | **0.005** | 1 | – | – |
| Low | 18 | 22.5% | | 1.920 | 1.0747–3.429 | **0.0276** |
| **EGFR Expression** | | | | | | |
| High | 62 | 53.7% | **0.018** | 1 | – | – |
| Low | 18 | 46.3% | | 1.714 | 1.0678–2.750 | – |
| **KRAS Mutation** | | | 0.2 | | | |
| High | 27 | 33.8% | | – | – | – |
| Low | 53 | 66.2% | | – | – | – |

a In univariate analyses, log-rank tests were conducted.
b In the multivariate Cox proportional hazard model, only variables with P < 0.15 in univariate analysis were included and the "enter method" was applied.
c Data on age of two patients were missing.
